# Lymphocyte Count and Neutrophil-to-Lymphocyte Ratio Are Associated with Mild Cognitive Impairment in Parkinson’s Disease: A Single-Center Longitudinal Study

**DOI:** 10.3390/jcm11195543

**Published:** 2022-09-22

**Authors:** Elena Contaldi, Luca Magistrelli, Marco Cosentino, Franca Marino, Cristoforo Comi

**Affiliations:** 1 Movement Disorders Centre, Neurology Unit, Department of Translational Medicine, University of Piemonte Orientale, 28100 Novara, Italy; 2 PhD Program in Medical Sciences and Biotechnology, University of Piemonte Orientale, 28100 Novara, Italy; 3 PhD Program in Clinical and Experimental Medicine and Medical Humanities, University of Insubria, 21100 Varese, Italy; 4 Center of Research in Medical Pharmacology, University of Insubria, 21100 Varese, Italy; 5 Center for Research in Neuroscience, University of Insubria, 21100 Varese, Italy; 6 Department of Translational Medicine, Neurology Unit, S. Andrea Hospital, University of Piemonte Orientale, 13100 Vercelli, Italy

**Keywords:** Parkinson’s disease, neutrophil-to-lymphocyte ratio, lymphocyte count, peripheral immune system, cognitive impairment

## Abstract

Lymphocyte count and neutrophil-to-lymphocyte ratio (NLR) may represent useful biomarkers of Parkinson’s disease (PD), but their role in PD-related mild cognitive impairment (MCI) has not been fully elucidated. The present study aimed to confirm whether these immunological measures can discriminate PD patients from healthy controls (HC) and establish their feasibility as prognostic biomarkers of MCI in PD. Immunological data at baseline were analyzed in 58 drug-naïve PD patients and 58 HC matched 1:1 for age, sex, and cardiovascular comorbidities. We selected a subgroup of 51 patients from this initial cohort who underwent longitudinal neuropsychological assessments through the Addenbrooke’s Cognitive Examination Revised (ACE-R) test. We considered the last examination available to analyze the relationship between ACE-R test scores and immunological measures. We found that lymphocyte count was lower and NLR higher in PD than HC (*p* = 0.006, *p* = 0.044), with AUC = 0.649 and 0.608, respectively. Secondly, in PD-MCI there were significantly higher levels of circulating lymphocytes (*p* = 0.002) and lower NLR (*p* = 0.020) than PD with normal cognitive status (PD-NC). Correlations between lymphocyte count and ACE-R total score and memory subitem (*r =* −0.382, *p* = 0.006; *r =* −0.362, *p* = 0.01), as well as between NLR and ACE-R total score and memory subitem (*r =* 0.325, *p* = 0.02; *r =* 0.374, *p* = 0.007), were also found. ROC curve analysis showed that lymphocyte count and NLR displayed acceptable discrimination power of PD-MCI with AUC = 0.759 and 0.691, respectively. In conclusion, we suggest that an altered peripheral immune phenotype could foster cognitive decline development in PD, thus opening the possibility of immune-targeting strategies to tackle this disabling non-motor feature.

## 1. Introduction

Parkinson’s disease (PD) is one of the most common neurodegenerative diseases. The clinical picture is characterized by motor symptoms, including bradykinesia, rigidity, tremor and postural instability, and a wide array of non-motor features [1]. The pathological hallmark of PD is represented by intraneuronal α-synuclein-positive inclusions called Lewy bodies and loss of dopaminergic neurons in the substantia nigra pars compact (SNc), the dorsal motor nucleus of the vagal nerve, the locus coeruleus, the pedunculopontine nucleus, and the nucleus basalis of Meynert [2]. Many pathogenic pathways, including endolysosomal and mitochondrial dysfunction, have been considered key factors. Moreover, a deeper understanding of the immune mechanisms involved in PD is being considered as well: recent evidence suggested that lower lymphocyte count was associated with an increased risk of subsequent PD diagnosis [3] and could predict ApoE ε4-related cognitive decline in PD [4]. In particular, T lymphocytes can be found in the brain of both postmortem human PD subjects and the 1-methyl-4-phenyl-1,2,3,6-tetrahydropyridine (MPTP) mouse model of PD [5], whereas reduced CD4+ T cells with increased levels of T helper (Th) 1 were observed in the peripheral blood of PD patients compared with healthy controls (HC) [6]. In the context of a disrupted immune network largely involving lymphocytes, a promptly available indicator of peripheral inflammation is represented by the neutrophil-to-lymphocyte ratio (NLR). NLR is based on two distinct but complementary leukocyte subpopulations and alterations of this index can be found in a wide variety of medical conditions such as cancer, inflammatory and cardiovascular diseases [7,8,9]. Even though controversial results have been reported and it is unclear whether the NLR can adequately reflect peripheral inflammation, several studies observed high NLR values in PD patients [10]. Furthermore, a connectometry analysis by Haghshomar and colleagues revealed in early PD significant negative correlations between NLR and white matter quantitative anisotropy in bilateral cingulum, body and left crus of fornix, body, and splenium of corpus callosum, the bilateral corticospinal tract, and the superior cerebellar peduncle [11]. Some of these structures, in particular the fornix, the corpus callosum, and the superior cerebellar peduncle, have been implicated in short- and long-term memory impairment in PD patients [12], but longitudinal evaluations assessing the contribution of peripheral immune mechanisms to cognitive impairment are still lacking.

Therefore, the present study aimed to elucidate whether (i) lymphocyte count and NLR can discriminate PD patients from HC; (ii) they may represent feasible prognostic biomarkers of mild cognitive impairment (MCI) in PD.

## 2. Materials and Methods

This study was carried out by following the ethical guidelines of the local Ethics Committee, and all patients gave their written informed consent (CE 65/16). Patients were recruited in the context of a study aiming to define the role of the peripheral immune system in PD progression conducted at the Movement Disorders Center of University Hospital Maggiore della Carità, Novara, Italy [6,13]. The database currently includes 70 drug-naïve PD patients enrolled in a longitudinal study and 94 HC. We considered as inclusion criteria subjects with an established clinical diagnosis of PD [14], aged between 45 and 80 years old, speaking Italian as their first language, and with adequate abilities to perform neuropsychological tests. Exclusion criteria for all subjects were brain abnormalities on magnetic resonance imaging tests, a history of chronic autoimmune diseases or cancer, and administration of immunomodulatory treatment. For the specific purpose of the present study, we excluded patients with dementia or severe depression at baseline and incomplete immunological data. Complete medical records and total leukocyte count with subpopulations (neutrophils, lymphocytes, monocytes, eosinophils, and basophils) measured in peripheral blood at baseline were analyzed. The NLR was calculated as absolute neutrophil count divided by absolute lymphocyte count. All patients were in the drug-naïve condition to exclude potential effects of anti-Parkinsonian treatment on the immunological profile [15,16]. At the time of enrollment, clinical examination was performed by neurologists with experience in movement disorders, and motor symptoms were assessed by using the Unified Parkinson’s Disease Rating Scale (UPDRS) part III and the Hoehn and Yahr (HY) scale [17,18]. To explore the usefulness of the lymphocyte count and NLR in identifying PD patients, available immunological data of HC matched 1:1 for age (±1 year), sex, and cardiovascular disease status, were examined. Thereafter, a subgroup of patients with longitudinal neuropsychological evaluation was selected from the initial PD cohort. Based on the last examination available, PD patients were divided into two groups based on cognitive scores assessed through Addenbrooke’s Cognitive Examination-Revised (ACE-R) test [19]. According to previous literature [20], a cut-off score of 89 was used to discriminate between PD-normal cognition (PD-NC) and PD with mild cognitive impairment (PD-MCI). Raw total and subitem scores were adjusted for age, sex, and education according to established correction grids [21].

Concerning statistical analysis, variables were expressed as counts and percentages when categorical and as mean ± standard deviation when continuous. The distribution and normality of data were assessed by using the Shapiro–Wilk test. Accordingly, differences between groups were analyzed through the *t*-test for independent samples after testing for homogeneity of variances (Levene statistics) or non-parametric Mann–Whitney U test/Kruskal–Wallis test. Comparisons between categorical variables were assessed by using Fisher’s exact test or Chi-square test as appropriate. A receiver operating characteristic (ROC) curve analysis was carried out to establish the discriminatory power of lymphocyte count and NLR between PD vs. HC and PD-NC vs. PD-MCI. The area under the curve (AUC) and significance values were obtained, and AUC values interpretation was determined according to previous literature [22]. Optimal cut-offs were chosen by coordinate tracing of the ROC curve according to Youden’s index analysis. Sensitivity, specificity, positive and negative likelihood ratios (LR+, LR−), and positive and negative predictive values (PPV, NPV) were computed. Spearman correlation analysis was carried out to find the relationship between ACE-R total and subitem scores and immunological data. The significance level was set to *p* < 0.05. All analyses were conducted by using SPSS Version 25 (IBM Corporation, Armonk, NY, USA).

## 3. Results

### 3.1. Lymphocyte Count and Neutrophil-to-Lymphocyte Ratio in Parkinson’s Disease vs. Healthy Subjects

At total of 58 PD patients and 58 matched HC were recruited. The demographic, clinical, and immunological characteristics of both groups are reported in Table 1. We found that the total number of lymphocytes was lower (*p* = 0.006) and the NLR higher (*p* = 0.044) in PD subjects, whereas no other statistically significant differences were found between groups.

**Figure 1:**
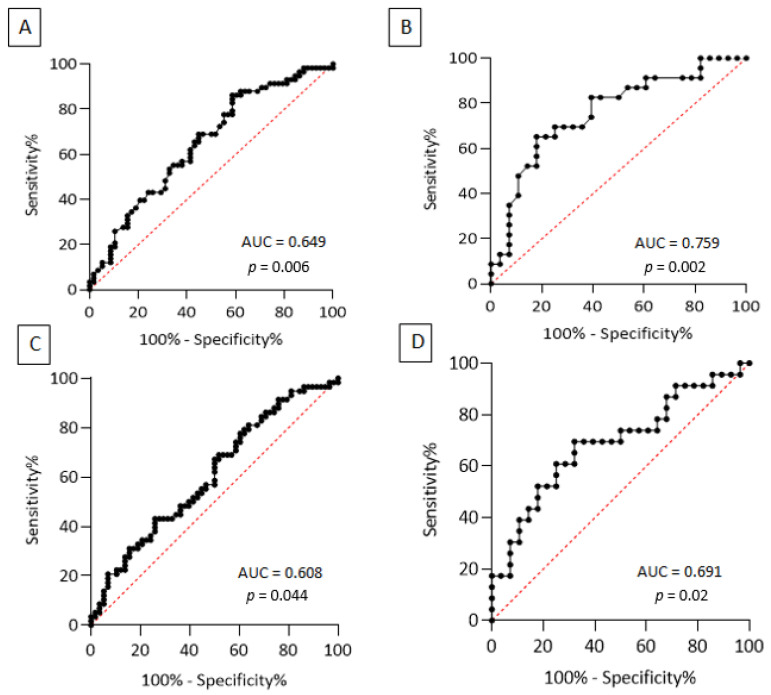
ROC curve of lymphocyte count to discriminate PD patients from HC (panel **A**) and PD-MCI from PD-NC (panel **B**); ROC curve of NLR values to discriminate PD patients from HC (panel **C**) and PD-MCI from PD-NC (panel **D**).

ROC curve analysis was therefore employed to detect the utility of these immunological measures in discriminating PD from HC, finding for lymphocyte count an AUC value = 0.649 (95% CI 0.0549–0.748, *p* = 0.006) and for NLR an AUC value = 0.608 (95% CI 0.506–0.711, *p* = 0.044) (see Figure 1 panel A and C). Regarding lymphocyte count, an optimal cut-off value ≤ 1.915 × 10^3^/microL had 69% sensitivity (95% CI 56.20–79.38%) and 55.2% specificity (95% CI 42.45–67.25%), whereas an optimal cut-off for NLR ≥ 2.065 showed a sensitivity of 69% (95% CI 56.20–79.38%) and a specificity of 48.3% (95% CI 35.93–60.84%), see Table 2.

### 3.2. Lymphocyte Count and Neutrophil-to-Lymphocyte Ratio in Parkinson’s Disease-Related Mild Cognitive Impairment

From the initial PD cohort, 51 subjects with longitudinal neuropsychological evaluation were then selected and divided into two groups according to cognitive status. Demographic, clinical, and immunological characteristics of PD-NC and PD-MCI are reported in Table 3.

**Figure 2:**
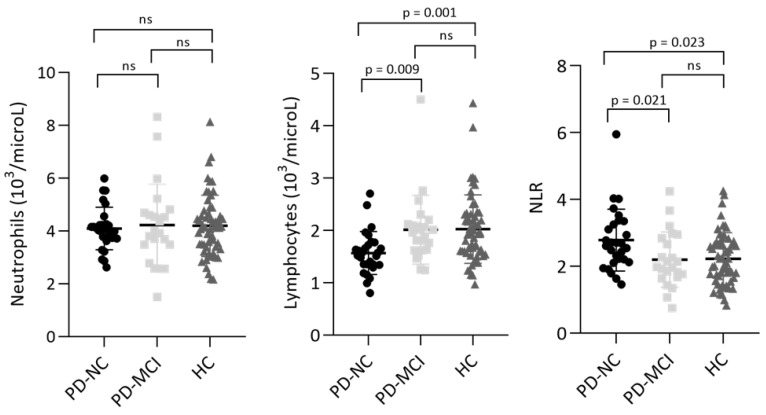
Kruskal–Wallis test with Bonferroni correction for levels of circulating neutrophils, lymphocytes, and NLR in PD and HC. Data are plotted as means and standard deviations.

PD-MCI reported significantly lower scores in ACE-R total, attention and orientation, memory, fluency, and visuospatial subitems. Regarding immunological parameters, in PD-MCI there were significantly higher levels of circulating lymphocytes (*p* = 0.002) and lower NLR values (*p* = 0.020). The comparison between PD-MCI, PD-NC, and HC using the Kruskal–Wallis test after Bonferroni correction is reported in Figure 2. ROC curve analysis was performed to establish whether lymphocyte count and NLR could discriminate between PD-MCI and PD-NC. PD-MCI were detected with acceptable AUC values by both lymphocyte count (0.759, 95% CI 0.625–0.894, *p* = 0.002) and NLR (0.691, 95% CI 0.542–0.840, *p* = 0.02) (see Figure 1 panel B and D). An optimal cut-off value for lymphocyte count ≥ 1.790 × 10^3^/microL had 65.2% sensitivity (95% CI 44.89–81.19%) and 82.1% specificity (95% CI 64.41–92.12%), whereas a cut-off value for NLR ≤ 2.295 showed a sensitivity of 69.6% (95% CI 49.13–84.4%) and a specificity of 67.8% (95% CI 49.34–82.07%) (see Table 2). Correlations between lymphocyte count and ACE-R total score and memory subitem (*r =* −0.382, *p* = 0.006; *r =* −0.362, *p* = 0.01) as well as between NLR and ACE-R total score and memory subitem (*r =* 0.325, *p* = 0.02; *r =* 0.374, *p* = 0.007), were also found, whereas no statistically significant correlations were observed between immunological measures and age at onset, sex, disease duration, levodopa equivalent daily dose (LEDD), and UPDRS-III score.

## 4. Discussion

The results of the present study highlight different profiles of peripheral immune cells in PD patients compared with HC and in relation to cognitive status. In more detail, we first found that PD subjects display significantly lower levels of circulating lymphocytes and higher NLR than HC. Secondly, we reported in PD-MCI higher levels of circulating lymphocytes and lower NLR than PD-NC.

Decreased levels of circulating lymphocytes in PD patients have been reported in several studies [6,10,23], and the feasibility of NLR as a biomarker has been explored as well. Akıl and colleagues [24] found that NLR values ≥ 2.25 resulted in 73% sensitivity and 74% specificity in identifying PD patients, determining higher predictive power than carcinoembryonic antigen (CEA). Similarly, another study [25] established a cut-off value of 2.39 with 65% sensitivity and 75% specificity (AUC = 0.714). Compared to these results, the present research showed for NLR lower AUC values and poorer prediction of PD diagnosis: however, it should be borne in mind that in our case-control design strict matching criteria, including cardiovascular comorbidities, were applied. Regarding the relationship between NLR and PD-related symptoms, another work [26] failed to find statistically significant differences in NLR between 13 akinetic-rigid and 33 tremor-dominant patients. Furthermore, no association with disease severity [10,27] and controversial results concerning the relationship with disease duration and LEDD [10,25,26] have also been described.

The disruption of peripheral immune mechanisms is involved in cognitive impairment as well. For example, in previous studies [28,29], lymphocyte levels were significantly decreased in Alzheimer’s disease (AD) and MCI patients compared with HC. Furthermore, altered levels of interleukin (IL)-10, IL-1β, IL-4, and IL-2 were reported in the MCI stage of dementia with Lewy bodies (DLB) and MCI-AD, thus supporting the role of the peripheral immune system early in the disease process [30]. In this context, a pro-inflammatory shift leading to higher NLR values was reported as an independent risk factor for MCI [28,31]. Intriguingly, high preoperative NLR values were also associated with cognitive dysfunction in patients undergoing carotid endarterectomy [32] and after acute ischemic stroke [33].

On the other hand, our results showed that PD-MCI display significantly lower NLR and higher levels of circulating lymphocytes than PD-NC, whereas no differences were found regarding other indexes of peripheral inflammation, such as erythrocyte sedimentation rate (ESR) and C-reactive protein (CRP). Moreover, ROC curve analysis showed that both lymphocyte count and NLR displayed acceptable discrimination power of PD-MCI with AUC = 0.759 and 0.691, respectively. Because the neutrophil count was only mildly elevated in PD-MCI, it is unequivocal that the strongest contribution in NLR values derives from the average number of total circulating lymphocytes (2.01 × 10^3^/microL in PD-MCI vs. 1.57 × 10^3^/microL in PD-NC). It should also be noted that both levels of circulating lymphocytes and NLR are almost identical in PD-MCI and HC (2.01 × 10^3^/microL vs. 2.02 × 10^3^/microL; 2.20 vs. 2.23. See Figure 2). The trend toward normal immunological values in PD-MCI certainly lowers the reliability of these biomarkers in discriminating PD-MCI from HC, but at the same time raises several intriguing observations. One possible explanation for this unexpected finding is that PD patients with more severe trajectories of cognitive deterioration display higher levels of circulating lymphocytes as a result of profound adjustments leading to altered lymphocyte subpopulations. Indeed, altered levels of peripheral CD4+, CD8+, CD3+, and CD4+/CD8+ have been previously reported in cognitively impaired PD patients [34], whereas another study [35] reported in patients with worse cognitive scores higher levels of activated T regulatory cells (Treg) and Th1 and lower resting Treg. As suggested by the latter study, it is conceivable that the dysregulation of the Treg and Th1 compartments may significantly increase the vulnerability to the development of cognitive impairment. Interestingly, we also found significant correlations between lymphocyte count/NLR and the ACE-R memory subscore: Berankova and colleagues [36] demonstrated that this subscore has 90% sensitivity and 46% specificity in predicting PD dementia (PDD). Therefore, it can be speculated that an imbalance of peripheral immune cells may be involved in memory deficits and associated with an increased risk of dementia. It should also be highlighted that even though α-synuclein pathology is the main substrate of PDD, coexistent tau and amyloid-β pathologies are common and independently contribute to the development of cognitive decline in PD [37]. Indeed, several lines of evidence reported that neutrophil-related markers in peripheral blood could predict a decline in executive function in mild AD patients [38], and neutrophil extracellular traps (NETs) inside the cortical vessels and parenchyma of AD patients were also observed [39]. The discovery of NETs in AD brains suggests their role in the exacerbation of neuroinflammatory mechanisms through vascular and parenchymal damage, but their involvement in synucleinopathies has not yet been fully understood.

In this context, the advantage of NLR comes from integrating the information of two leukocyte subtypes, as altered lymphocyte levels express the impairment of regulatory pathways, whereas elevated neutrophils have been associated with increased oxidative stress and peripheral cytokine release [40]. Moreover, it overcomes the limits of absolute values of a single leukocyte subtype (which can be influenced by several factors), resulting in higher clinical significance compared with other inflammatory biomarkers [41].

To the best of our knowledge, this study demonstrates for the first time the relationship between established and readily available measures of peripheral inflammation (lymphocyte count and NLR) and the impairment of specific cognitive domains in PD-MCI. One strength of this research is represented by the careful selection of patients who were drug-naïve concerning anti-Parkinsonian treatment at baseline immunological assessment, thus excluding the potential interference of dopaminergic therapy. Moreover, when evaluating the discrimination power for lymphocyte count and NLR of PD and PD-MCI, we thoroughly considered medical conditions potentially affecting immunological measures. Some limitations should be mentioned as well, such as the small sample size and the relatively limited time of longitudinal analysis (3.14 ± 1.56 years). Furthermore, the diagnosis of PD-MCI was established through an abbreviated assessment, thus providing less diagnostic certainty than extensive neuropsychological test batteries [42]. Nonetheless, we suggest that future studies with prospective designs and larger cohorts should analyze the association between levels of circulating lymphocytes/NLR and MCI in early PD and explore their accuracy as biomarkers of PD progression.

## 5. Conclusions

Our study, though exploratory in nature, suggests that PD-MCI patients display an altered peripheral immune phenotype characterized by increased levels of circulating lymphocytes and reduced NLR. Whether this peculiar immunological profile could make patients more susceptible to cognitive decline development has yet to be fully clarified. The answer to this question could be of great interest, especially in the emerging scene of immune-targeting strategies in PD.

## Figures and Tables

**Table 1:** Demographic, clinical, and immunological characteristics of PD patients and healthy controls.

Variables	PD (*n* = 58)	HC (*n* = 58)	*p*-Value
Age, years	69.28 (8.13)	69.31 (8.18)	0.969
Sex, M/F	42/16	42/16	1.0
History of cardiovascular diseases	34 (58.6)	31 (53.4)	0.575
Disease duration, years	1.38 (0.91)	-	-
UPDRS-III “OFF”, score	13.62 (6.79)	-	-
H&Y stage -Stage 1 -Stage 2	41 (70.7) 17 (29.3)	-	-
Tremor dominant phenotype	39 (67.2)	-	-
ACE-R total score (baseline)	93.20 (2.92)	-	-
ACE-R attention and orientation (baseline)	18 (2.24)	-	-
ACE-R memory (baseline)	25.86 (4.87)	-	-
ACE-R fluency (baseline)	11.71 (2.80)	-	-
ACE-R language (baseline)	27.37 (2.86)	-	-
ACE-R visuospatial (baseline)	16.08 (2.49)	-	-
WBC (10^3^/microL)	6.47 (1.34)	6.71 (1.54)	0.600
RBC (10^6^/microL)	4.69 (0.50)	4.91 (0.78)	0.251
Hemoglobin (g/dL)	14.12 (1.40)	14.24 (1.13)	0.519
Hematocrit (%)	42.39 (4.02)	42.90 (3.38)	0.467
MCV (fL)	90.62 (3.30)	91.47 (4.93)	0.588
MCH (pg)	30.15 (1.10)	30.47 (1.73)	0.118
MCHC (g/dL)	33.87 (4.44)	33.52 (2.28)	0.601
Platelets (10^3^/microL)	218.75 (51.48)	222.63 (52.80)	0.612
Monocytes (10^3^/microL)	0.55 (0.17)	0.56 (0.15)	0.712
Eosinophils (10^3^/microL)	0.15 (0.09)	0.22 (0.21)	0.108
Basophils (10^3^/microL)	0.04 (0.03)	0.04 (0.02)	0.601
Neutrophils (10^3^/microL)	4.20 (1.22)	4.19 (1.16)	0.943
Lymphocytes (10^3^/microL)	1.73 (0.56)	2.02 (0.65)	*0.006*
NLR	2.63 (1.15)	2.23 (0.78)	*0.044*
ESR (mm/h)	12.14 (10.98)	-	-
CRP (mg/dL)	0.37 (0.97)	-	-

Abbreviations: ACE-R, Addenbrooke’s Cognitive Examination Revised; H&Y, Hoehn and Yahr; UPDRS, Unified Parkinson’s Disease Rating Scale; WBC, white blood cells; RBC, red blood cells; MCV, mean corpuscular volume; MCH, mean corpuscular hemoglobin; MCHC, mean corpuscular hemoglobin concentration; NLR, neutrophil-to-lymphocyte ratio; ESR, erythrocyte sedimentation rate; CRP, C-reactive protein. Variables are expressed as mean (SD) when continuous and counts (percentage) when categorical. Significant *p*-values are highlighted in italics.

**Table 2:** Cut-offs of lymphocyte count and NLR to discriminate between PD vs. HC and PD-NC vs. PD-MCI, with sensitivity, specificity, positive and negative likelihood ratios, and positive and negative predictive values.

Lymphocyte Count Cut-off Values	Sensitivity (95% CI)	Specificity (95% CI)	LR+	LR−	PPV	NPV
PD vs. HC						
≤1.915 (10^3^/microL)	69% (56.20–79.38%)	55.2% (42.45–67.25%)	1.54	0.56	60.6%	64%
PD-MCI vs. PD-NC						
≥1.790 (10^3^/microL)	65.2% (44.89–81.19%)	82.1% (64.41–92.12%)	3.64	0.42	75%	74.2%
**NLR Cut-off Values**	**Sensitivity (95% CI)**	**Specificity (95% CI)**	**LR+**	**LR−**	**PPV**	**NPV**
PD vs. HC						
≥2.065	69% (56.20–79.38%)	48.3% (35.93–60.84%)	1.33	0.64	57.1%	60.9%
PD-MCI vs. PD-NC						
≤2.295	69.6% (49.13–84.4%)	67.8% (49.34–82.07%)	2.16	0.45	64%	73.1%

Abbreviations: NLR, neutrophil-to-lymphocyte ratio; LR+, positive likelihood ratio; LR−, negative likelihood ratio; PD-MCI, PD with cognitive impairment; PD-NC, PD with normal cognition; PPV, positive predictive value; NPV, negative predictive value.

**Table 3:** Demographic, clinical, and baseline immunological characteristics of PD-NC and PD-MCI subgroups.

Variables	PD-NC (*n* = 28)	PD-MCI (*n* = 23)	*p*-Value
Age at baseline, years	67.39 (9.09)	68.80 (8.48)	0.185
Sex, M/F	18/10	18/5	0.360
Scholarity, years	10.86 (4.02)	9.17 (4.72)	0.116
History of cardiovascular diseases	17 (60.71)	13 (56.52)	0.783
Disease duration, years	2.86 (1.48)	3.48 (1.65)	0.162
UPDRS-III “ON”, score	13 (5.48)	13.65 (7.15)	0.753
LEDD, mg/day	360.84 (179.36)	409.65 (258.24)	0.463
H&Y stage -Stage 1 -Stage 2	20 (71.43) 8 (28.57)	18 (78.26) 5 (21.73)	0.749
Tremor dominant phenotype	19 (67.85)	15 (65.22)	1.0
ACE-R total score	92.67 (3.40)	80.15 (7.84)	*<0.0001*
ACE-R attention and orientation	18.13 (0.54)	20.06 (15.04)	*0.001*
ACE-R memory	26.40 (2.08)	21.57 (3.25)	*<0.0001*
ACE-R fluency	11.63 (2.22)	9.34 (2.42)	*<0.0001*
ACE-R language	27.35 (1.26)	26.04 (3.05)	0.117
ACE-R visuospatial	16.04 (1.24)	14.17 (1.91)	*<0.0001*
WBC (10^3^/microL)	5.95 (1.01)	7.07 (1.47)	*0.011*
RBC (10^6^/microL)	4.68 (0.44)	4.67 (0.59)	0.541
Hemoglobin (g/dL)	14.12 (1.30)	13.98 (1.58)	0.40
Hematocrit (%)	42.34 (3.54)	42.27 (4.76)	0.483
MCV (fL)	90.77 (2.84)	90.66 (3.73)	0.960
MCH (pg)	30.26 (1.02)	29.97 (1.23)	0.416
MCHC (g/dL)	34.48 (6.17)	33.09 (1.02)	0.405
Platelets (10^3^/microL)	214.15 (41.74)	223.35 (62.82)	0.841
Monocytes (10^3^/microL)	0.54 (0.18)	0.57 (0.16)	0.351
Eosinophils (10^3^/microL)	0.16 (0.09)	0.15 (0.09)	0.673
Basophils (10^3^/microL)	0.04 (0.02)	0.05 (0.03)	0.150
Neutrophils (10^3^/microL)	4.09 (0.80)	4.22 (1.55)	1.0
Lymphocytes (10^3^/microL)	1.57 (0.41)	2.01 (0.66)	*0.002*
NLR	2.78 (0.93)	2.20 (0.83)	*0.020*
ESR (mm/h)	10.50 (7.53)	10.65 (7.22)	0.857
CRP (mg/dL)	0.19 (0.22)	0.47 (1.28)	0.595

Abbreviations: ACE-R, Addenbrooke’s Cognitive Examination Revised; H&Y, Hoehn and Yahr; UPDRS, Unified Parkinson’s Disease Rating Scale; LEDD, levodopa equivalent daily dose; WBC, white blood cells; RBC, red blood cells; MCV, mean corpuscular volume; MCH, mean corpuscular hemoglobin; MCHC, mean corpuscular hemoglobin concentration; NLR, neutrophil-to-lymphocyte ratio; ESR, erythrocyte sedimentation rate; CRP, C-reactive protein. Variables are expressed as mean (SD) when continuous and counts (percentage) when categorical. Significant *p*-values are highlighted in italics.

## Data Availability

Anonymized data can be obtained from qualified researchers upon reasonable request to the corresponding author.
